# Hydrophilic Modification of Gadolinium Oxide by Building Double Molecular Structures

**DOI:** 10.3390/nano15181421

**Published:** 2025-09-16

**Authors:** Qin Li, Jian Chen, Xingwu Zhang, Chenjie Ruan, Weiwei Wu

**Affiliations:** 1 Yangjiang Nuclear Power Co., Ltd., Yangjiang 529941, China; 18506618870@163.com (Q.L.); chenjian_07@163.com (J.C.); zhangxingwu2025@163.com (X.Z.); powerjay03@163.com (C.R.); 2 School of Information Mechanics and Sensing Engineering, Xidian University, Xi’an 710126, China

**Keywords:** Gd_2_O_3_ nanoparticle, surface modification, hydrophilic modification, polyether amine

## Abstract

With the rapid growth of nuclear energy, effective shielding of radioactive nuclear by-products is critical for safety and environmental protection. Gadolinium (Gd) is ideal for neutron shielding due to its exceptionally high thermal neutron capture cross-section. Despite significant progress in developing various Gd-based shielding materials, poor interfacial compatibility between Gd_2_O_3_ and polymer matrices remains a significant limitation. In this study, we addressed this challenge by successfully modifying Gd_2_O_3_ nanoparticles (Gd_2_O_3_@SIT-M) through the construction of a dual-layer molecular coating using electrostatic interactions. Initially, Gd_2_O_3_ was functionalized with the silane coupling agent 3-(trihydroxysilyl) propyl-1-propane-sulfonic acid (SIT), followed by subsequent assembly of polyether amine M2070 onto this modified surface. The combined presence of hydrophilic sulfonic acid groups from SIT and amine-ether groups from M2070 endowed Gd_2_O_3_@SIT-M nanoparticles with excellent hydrophilicity, significantly reducing their aqueous contact angle to 14.34°. Consequently, this modification strategy notably enhanced the dispersion stability of Gd_2_O_3_ nanoparticles in aqueous solutions and polymer matrices. The developed approach thus provides an effective pathway for fabricating advanced polymer-based neutron shielding materials with improved dispersibility, stability, and overall performance.

## 1. Introduction

In recent decades, the extensive utilization of nuclear energy has significantly enhanced global energy supply, addressing the persistent challenge of balancing energy demands with environmental concerns. Currently, nuclear power provides approximately 10% of global electricity generation, substantially reducing greenhouse gas emissions compared to traditional fossil fuel-based sources [1,2]. As a sustainable and environmentally friendly alternative, nuclear energy thus occupies an increasingly vital position in modern energy strategies. Nevertheless, despite its notable benefits, the widespread application of nuclear energy is hindered by several critical issues, particularly the generation of radioactive byproducts during nuclear reactions. These byproducts emit various types of radiation, including alpha rays, beta rays, gamma rays, and neutrons [3,4,5,6]. Among these radiation types, neutron radiation has attracted significant attention due to its highly penetrating nature and substantial capability to cause irradiation damage, presenting a major challenge in radiation shielding [4,7].

A promising strategy to enhance neutron shielding performance involves incorporating elements with large neutron capture cross-sections into protective materials. This approach significantly improves both the neutron absorption capacity and mechanical properties of shielding composites. The rare-earth element gadolinium (Gd), recognized for its exceptional neutron capture efficiency since its discovery by Dunning et al. in 1935 [8], has consistently been validated as highly effective for neutron shielding applications in subsequent research [9,10,11]. Specifically, the isotope ^157^Gd possesses an extraordinarily high neutron capture cross-section of approximately 254,000 barns, which is about 67 times greater than the widely utilized neutron absorber, boron-10 (^10^B), a commonly used neutron absorber with a cross-section of approximately 3800 barns [9,12]. Other materials, such as cadmium (Cd) with a cross-section of 2450 barns and samarium (Sm) with around 5900 barns for ^149^Sm, also offer neutron absorption but fall short of Gd’s efficiency [13]. Additionally, while boron-based compounds like B_4_C are widely used, they are less effective against thermal neutrons compared to Gd_2_O_3_, and their incorporation into polymers often results in brittleness. Cadmium, although effective, poses environmental and health concerns due to its toxicity, making it less favorable for widespread use [14]. Moreover, Gd_2_O_3_ offers practical advantages over these alternatives. Unlike lead (Pb) or tungsten (W), which are effective against gamma and X-rays but poor neutron absorbers, Gd_2_O_3_ specifically targets neutron shielding. Consequently, gadolinium-based materials have been intensively explored as efficient neutron shielding candidates. Burke et al. [15], for instance, demonstrated that Gd_2_O_3_ coatings significantly improved radiation shielding performance through effective neutron attenuation and absorption.

Polymeric materials, including epoxy resin (ER), polyvinyl alcohol (PVA), and polyethylene (PE), have also emerged as attractive candidates for neutron shielding due to their capacity to moderate and scatter fast neutrons effectively [16]. However, their intrinsic thermal neutron shielding capabilities remain relatively limited. Thus, incorporating gadolinium compounds, particularly Gd_2_O_3_, into polymer matrices is a strategic approach to substantially enhance their neutron shielding effectiveness. Despite their potential, gadolinium-based polymer composites often encounter significant challenges related to poor interfacial compatibility between inorganic Gd_2_O_3_ fillers and organic polymer matrices, resulting in aggregation, poor dispersion stability, and diminished overall performance. For instance, Li et al. [17] revealed that nano-sized Gd_2_O_3_ particles demonstrated superior X-/γ-ray shielding performance compared to their micro-sized counterparts when incorporated into epoxy resins, attributable to their finer dispersion and smaller particle sizes. Nonetheless, the advantages of nanoscale particles diminished with increasing filler loading. Similarly, He et al. [18] reported that excessive nano-Gd_2_O_3_ incorporation adversely affected the thermal stability and mechanical properties of salicylic acid polyphenylene sulfide (SAPPS) composites. Furthermore, Huo et al. [19] indicated that surface-modified Gd_2_O_3_ particles significantly improved both thermal stability and mechanical properties in high-density polyethylene (HDPE) composites, demonstrating that surface modification is critical to addressing interfacial incompatibility and enhancing shielding performance.

Given the importance of effective interfacial interactions for achieving uniform dispersion and stable composite structures, this study aims to enhance the dispersibility and compatibility of Gd_2_O_3_ nanoparticles within aqueous and polymer matrices. Specifically, we introduced a two-step hydrophilic surface modification strategy involving an initial functionalization with organosilane SIT, followed by the assembly of a secondary layer through electrostatic interactions between negatively charged sulfonic acid groups from SIT and positively charged amino groups from polyether amine M2070. This bilayer modification significantly improved the aqueous stability and dispersion uniformity of Gd_2_O_3_ nanoparticles, ultimately providing an efficient pathway toward high-performance polymer-based neutron shielding materials with optimized thermal, mechanical, and neutron attenuation properties.

## 2. Materials and Methods

Materials: 3-(trihydroxy silyl)propyl-1-propane-sulfonic acid (SIT, C_3_H_10_O_6_SSi, 30–35% in water), Sodium hydroxide (NaOH, 98%), γ-aminopropyl triethoxysilane (KH-550, 99%) and γ-(2,3-epoxypropylene oxide) propyltrimethoxysilane (KH-560, 99%) was purchased from Macklin (Shanghai Macklin Biochemical Co., Ltd., Shanghai, China) and used as received. Type 732 strong acidic styrene cation-exchange resin with sulfonic acid groups was purchased from Aladdin (Shanghai Aladdin Bio-Chem Technology Co., Ltd., Shanghai, China). Polyetheramine (M2070, Mn∼2000, >98%) was bought from Suzhou Long Branch Industrial Co., Ltd. (Suzhou, China) Styrene cation exchange resin (Amberlite 732) was purchased from Aladdin (Melbourne, Australia). All other chemicals such as methanol and ethanol were of analytical grade and were used without further purification. Deionized water was used in the experiment.

Synthesis of Gd_2_O_3_@SIT: 0.5 g Gd_2_O_3_ nanoparticles (Gd_2_O_3_ NPs) were dispersed in 20 mL deionized water and ultrasound for 30 min to form a milky white Gd_2_O_3_ aqueous solution. Then, 1 g SIT was added to the DI water and then the pH was adjusted to 6–7 with 1 mol/L NaOH, which was mixed with the previous Gd_2_O_3_ aqueous solution and stirred for 12 h at room temperature. The product was dialyzed (a dialysis bag with 5000 molecular) for 12 h to remove the residual SIT during which the fresh DI water was altered every four hours. The suspension was added to the pre-treated 732 Styrene cation exchange resin with stirring at room temperature for 24 h to exchange the Na-type with the H-type. The obtained Gd_2_O_3_@SIT nanoparticles were filtered in DI water for future use.

Synthesis of Gd_2_O_3_@SIT-M: Gd_2_O_3_@SIT nanoparticles (0.5 g) were first dispersed in 20 mL aqueous solvent and sonicated for 10 min. Then, Polyether amine M2070 aqueous solution was drop-wise added to the above suspension until the pH reached 7 with constant stirring. The resulting solution was then dried on a hot plate fixed at 80 °C with stirring at 150 rpm/min and then kept at 50 °C under vacuum to obtain the Gd_2_O_3_@SIT-M sample.

Pretreatment process of cation exchange resin: The conditioning of the resin before the preconcentration procedure was conducted in order to remove any metal impurities. For this purpose, type 732 cation-exchange resin was successively washed with deionized water, 5% HCl solution, and deionized water.

Measurements: The attenuated total reflection infrared (ATR-IR) spectroscopy was conducted by a smart ATR-IR spectrometer (NicoletiS10, Thermo Fisher Scientific, Waltham, MA, USA) equipped with a smart OMNI reflection ranging from 400 to 4000 cm^−1^. Zeta potentials of samples were analyzed by a zeta potential analyzer (Brookhaven Instruments, Nashua, USA). Contact angle measurements were performed using a contact angle goniometer (Theta Flex, Biolin, Finland). Thermo-gravimetric analysis (STA 449F5, Netzsch, Germany) experiments were carried out under a N_2_ atmosphere with a heating rate of 5 °C/min. XRD patterns were recorded on an X-ray diffractometer (D8 ADVANCE, Bruker, Germany) focus advanced X-ray diffractometer operated at 40 kV and 40 mA using a Cu Ka radiation of wavelength (λ = 1.5406 Å). The equipment was calibrated by a standard silicon sample before measurement. Scanning electron microscopy (SEM) images were obtained from a field-emission scanning electron microscopy (FESEM, Apreo HiVac, FEI, Hillsboro, USA), at an accelerating voltage of 5 kV. Samples were dispersed in aqueous solution, attached on the conductive carbon adhesive tape, dried in a vacuum oven and then sputter-coated with 5 nm gold before observation. Transmission electron microscope (TEM, JEM-2100F, JEOL, Tokyo, Japan) images were obtained by evaporating a drop of aqueous dispersion of samples on carbon-coated copper grids, followed by measurement on a Titan TEM operating at 120 kV.

## 3. Results

**Figure 1:**
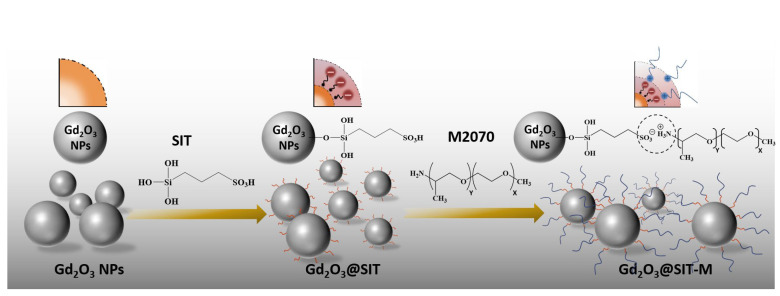
Schematic diagram of hydrophilic modification constructed by double molecular layer structure.

As illustrated in the schematic diagram of Figure 1, the hydrophilic modification of Gd_2_O_3_ nanoparticles is achieved via a two-step surface modification process. Initially, Gd_2_O_3_ particles are functionalized with the organosilane SIT, forming a stable intermediate layer through covalent bonding between the surface hydroxyl groups of Gd_2_O_3_ and silanol groups from SIT molecules. Subsequently, an additional polyether amine (M2070) layer is constructed onto the Gd_2_O_3_@SIT through electrostatic interactions between negatively charged sulfonic acid groups (–SO_3_H) of SIT and positively charged amine groups of M2070, resulting in a robust double molecular layer.

**Figure 2:**
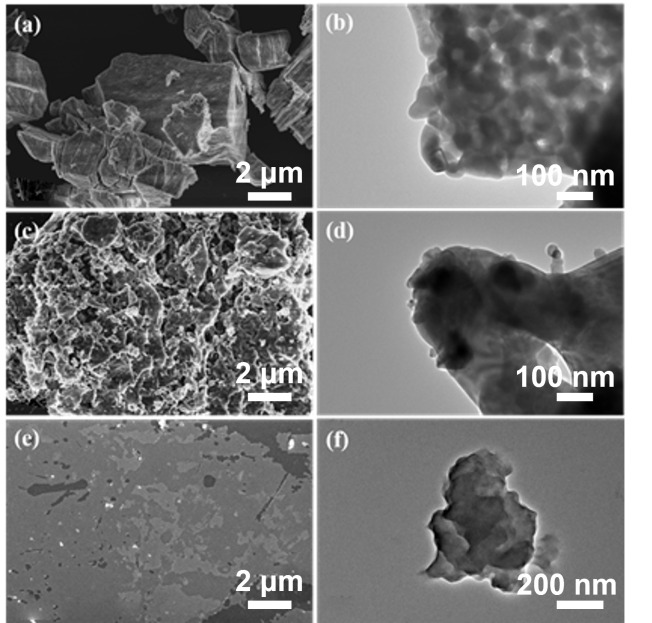
(**a**) SEM image and (**b**) TEM image of Gd_2_O_3_, (**c**) SEM image and (**d**) TEM image of Gd_2_O_3_@SIT, (**e**) SEM image and (**f**) TEM image of Gd_2_O_3_@SIT-M, respectively.

To systematically investigate the micromorphology evolution associated with each modification stage, pristine Gd_2_O_3_, Gd_2_O_3_@SIT, and Gd_2_O_3_@SIT-M samples were characterized by scanning electron microscopy (SEM) and transmission electron microscopy (TEM), as presented in Figure 2. As depicted in Figure 2a,b, pristine Gd_2_O_3_ particles exhibit clearly defined surfaces without the presence of any organic layers. In contrast, Figure 2c,d distinctly demonstrate the formation of a noticeable organic SIT layer covering the Gd_2_O_3_ surface after initial functionalization, significantly altering its surface texture and morphology. Furthermore, (Figure 2e,f) present SEM and TEM images of Gd_2_O_3_@SIT-M, revealing substantial lamellar structures and confirming an abundant and uniform distribution of the outer polyether amine layer (M2070). It is particularly noteworthy that the dual-layer organic modification plays a crucial role in enhancing the dispersibility and stability of Gd_2_O_3_ nanoparticles within aqueous solutions or polymer matrices. These improvements are anticipated to advance their applications as functional fillers for the development of novel neutron-shielding composite materials.

In the bright-field TEM image of Figure S1, the sample appears as an agglomerate of sub-micrometer primary particles with discernible interparticle boundaries. The corresponding EDS maps show that Gd and O are strongly co-localized across the entire particle body, confirming that the inorganic domains are Gd–O rich and consistent with Gd_2_O_3_. No segregated Gd-free or O-free regions are observed at the mapping resolution, indicating the absence of detectable secondary inorganic phases within the probed area. A weak but discernible Si signal is preferentially distributed along the outer rim of the agglomerate, suggesting a thin Si–O–containing surface layer at the particle periphery. This peripheral enrichment is consistent with the intended surface functionalization route that introduces Si-bearing moieties from SIT and does not alter the Gd–O-dominated core. The C map appears nearly uniform over the field of view; this is expected because it primarily originates from the supporting carbon film and, potentially, the organic surface ligands. Consequently, the C signal is not diagnostic of the inorganic core composition.

**Figure 3:**
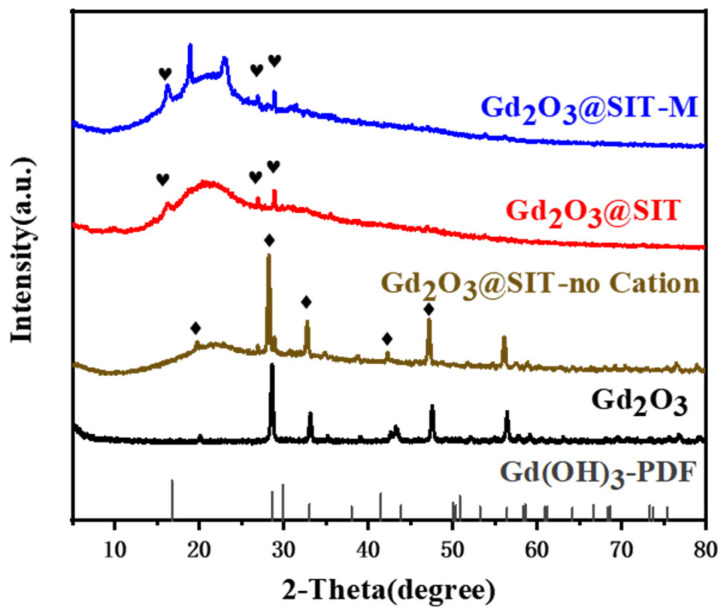
XRD patterns of Gd_2_O_3_, Gd_2_O_3_@SIT and Gd_2_O_3_@SIT-M. Heart symboles represent the dominant diffraction angles of Gd(OH)_3_ and the diamond symboles represent the dominant diffraction angle of Gd_2_O_3_.

Subsequently, XRD analysis was performed on Gd_2_O_3_@SIT-M to elucidate its structural evolution. As presented in Figure 3, characteristic diffraction peaks observed at approximately 16.3°, 26.9°, and 28.9° can be attributed to the presence of Gd(OH)_3_ phases [20]. To investigate the underlying cause of this physical phase transition, we also conducted comparative XRD analyses on samples modified individually with SIT, KH550, and KH560 (Figures S2–S4). These analyses produced nearly identical XRD patterns, indicating no detectable alteration in the crystal structure of Gd_2_O_3_ before ion-exchange treatment. However, after passing through the cation exchange resin, all modified samples uniformly exhibited a phase conversion to Gd(OH)_3_. The detailed mechanisms responsible for this phenomenon will be addressed comprehensively in our subsequent research. Additionally, upon the introduction of the polyether amine M2070, two new characteristic peaks emerged near 20°, as highlighted in Figure 3. These peaks likely originate from the formation of novel chelation complexes between M2070 and the functionalized surface of Gd_2_O_3_@SIT, a hypothesis that warrants further investigation.

In addition, we further used the Rietveld refinement to calculate the lattice parameters. As shown in Figure S5 and Table S1, The crystallographic structures of the obtained samples were analyzed by Rietveld refinement of the powder X-ray diffraction (XRD) patterns, consistent with JCPDS card No. 86-2477 for Gd_2_O_3_ and JCPDS card No. 83-2037 for Gd(OH)_3_ [20]. The quality of the refinement was evaluated by the profile residual (Rₚ), the weighted profile residual (R_wp_), and the goodness-of-fit indicator (χ^2^). The low values of Rₚ and R_wp_, and χ^2^ indicate a good agreement between the experimental and calculated diffraction patterns.

For sample 1 (Figure S5a), the diffraction peaks were sharp and intense, characteristic of a highly crystalline structure. The refined lattice parameters were a = b = c = 10.8099 Å, in good agreement with reported values for cubic system for Gd_2_O_3_. After acid treatment, the lattice parameters of the sample 2 exhibit noticeable changes, several characteristic reflections of Gd_2_O_3_ disappear or decrease in intensity, while new peaks emerge that can be assigned to hexagonal Gd(OH)_3_. The refined lattice parameters of Gd(OH)_3_ (a = b = 6.3291 Å, c = 3.6126 Å) are consistent with reported values, further validating the phase identification. The specific lattice parameters were summarized in Table S1.

**Figure 4:**
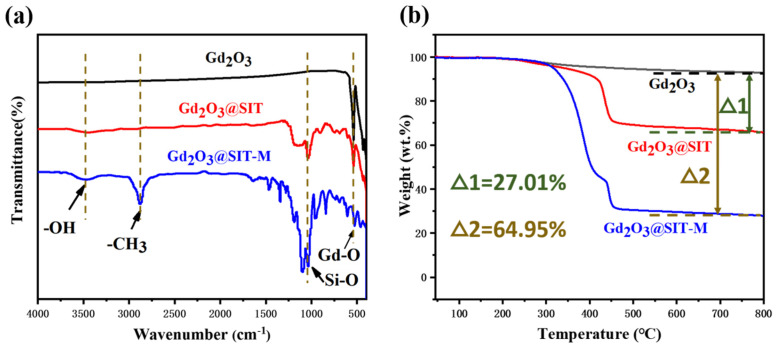
(**a**) ATR-IR spectroscopy of Gd_2_O_3_, Gd_2_O_3_@SIT and Gd_2_O_3_@SIT-M, (**b**) TGA traces of Gd_2_O_3_, Gd_2_O_3_@SIT and Gd_2_O_3_@SIT-M under N_2_ atmosphere at 5 °C/min.

Figure 4a presents the attenuated total reflection infrared (ATR-IR) spectra of pristine Gd_2_O_3_, Gd_2_O_3_@SIT, and Gd_2_O_3_@SIT-M samples. In the spectrum of Gd_2_O_3_@SIT-M, the prominent absorption band at approximately 544 cm^−1^ is attributed to the characteristic Gd–O vibrational modes of the inorganic core [15]. Additionally, the broad bands between 3500 and 2500 cm^−1^ correspond to the C–H stretching vibrations originating from the polymer backbone of the polyether amine (M2070) [21]. Distinct absorption peaks observed at 1311 cm^−1^ and 1168 cm^−1^ can be assigned to the symmetric and asymmetric stretching vibrations of sulfonate groups (O=S=O), respectively, confirming the successful modification by SIT molecules [22]. Furthermore, the intense peak at approximately 1060 cm^−1^ is characteristic of the asymmetric stretching vibration mode of Si–O–Si bonds from the SIT layer. The strong, broad band observed around 3470 cm^−1^ indicates the presence of O–H stretching and bending vibrations, which can likely be attributed to hydroxyl groups formed as a result of the cation exchange resin treatment, causing partial conversion of Gd_2_O_3_ to Gd(OH)_3_ [23].

Figure 4b displays the thermogravimetric analysis (TGA) curves for pristine Gd_2_O_3_, Gd_2_O_3_@SIT, and Gd_2_O_3_@SIT-M under a nitrogen atmosphere with a heating rate of 5 °C/min. It is notable that neither Gd_2_O_3_@SIT nor Gd_2_O_3_@SIT-M exhibited any discernible weight loss below 200 °C, indicating the absence of residual aqueous solvents and confirming their negligible volatility. As the temperature increased beyond 250 °C, decomposition of the organic molecular layers began, with Gd_2_O_3_@SIT-M displaying a higher decomposition rate compared to Gd_2_O_3_@SIT. This accelerated decomposition can be attributed to the comparatively weaker thermal stability of the polyether amine (M2070) polymer chains. A plateau occurring around 430 °C in the TGA curve of Gd_2_O_3_@SIT-M reflects the distinct thermolysis temperatures of the bilayer organic structure, implying stepwise decomposition of the SIT and M2070 layers. Upon further increasing the temperature to 800 °C, the residual weight percentages of Gd_2_O_3_@SIT and Gd_2_O_3_@SIT-M were determined as 66.01 wt.% and 28.06 wt.%, respectively. This indicates that the dual molecular organic layers in Gd_2_O_3_@SIT-M constituted approximately 64.95 wt.% of the total mass, thereby significantly enhancing its hydrophilic character and dispersibility.

**Figure 5:**
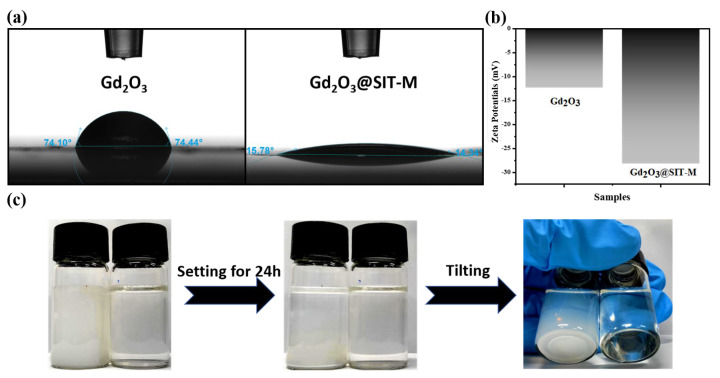
(**a**) Contact angles of Gd_2_O_3_ and Gd_2_O_3_@SIT-M, (**b**) Zeta potentials of Gd_2_O_3_ and Gd_2_O_3_@SIT-M in aqueous solution, (**c**) Photographs of aqueous stabilities of 10.0 mg/mL of Gd_2_O_3_ (left) and Gd_2_O_3_@SIT-M (right).

As illustrated in Figure 5a, the measured contact angles of pristine Gd_2_O_3_ and modified Gd_2_O_3_@SIT-M were 74.10° and 14.34°, respectively. This significant reduction in the contact angle indicates a marked enhancement in the hydrophilicity of Gd_2_O_3_, attributable to the successful construction of a bilayer organic coating on its surface. To quantitatively assess the stability of these particles in aqueous media, zeta potential measurements were performed. As depicted in Figure 5b, the zeta potential of Gd_2_O_3_@SIT-M (−28.2 mV) is substantially lower compared to that of pristine Gd_2_O_3_ (−12.36 mV). This enhanced negative surface charge arises from the introduction of the hydrophilic organic layers, which effectively increases surface wettability and electrostatic repulsion among particles, thus improving their aqueous dispersibility and stability.

To further confirm dispersion stability, aqueous dispersions with a concentration of 10.0 mg/mL for both pristine Gd_2_O_3_ and Gd_2_O_3_@SIT-M were prepared and allowed to stand undisturbed at room temperature for 24 h. As shown in Figure 5c, visible sedimentation occurred in the pristine Gd_2_O_3_ suspension, while no precipitation or agglomeration was observed in the Gd_2_O_3_@SIT-M dispersion, even upon tilting. This clearly demonstrates that the modified Gd_2_O_3_@SIT-M possesses superior colloidal stability in aqueous environments compared to unmodified Gd_2_O_3_. Furthermore, as shown in Supplementary Figure S6, after storage at room temperature for three months, Gd_2_O_3_@SIT-M remained dispersible and stable in an aqueous medium for at least 24 h without observable sedimentation. Collectively, these results conclusively illustrate that the electrostatically assembled hydrophilic double-layer structure formed on the Gd_2_O_3_ surface effectively prevents particle aggregation and sedimentation, resulting in stable and uniformly dispersed aqueous suspensions suitable for long-term practical applications.

## 4. Conclusions

In summary, hydrophilically modified Gd_2_O_3_ nanoparticles coated with double organic molecular layers were successfully fabricated through a two-step modification process involving the organosilane SIT and the polyether amine M2070. The initial modification step involved covalent bonding between hydroxyl groups on the Gd_2_O_3_ surface and the silanol groups of SIT, creating an intermediate surface layer with negatively charged sulfonic acid groups. Subsequently, electrostatic interactions between these negatively charged sulfonic acid groups on Gd_2_O_3_@SIT and positively charged amino groups on M2070 facilitated the formation of a stable and robust bilayer structure, referred to as Gd_2_O_3_@SIT-M. Characterization results confirmed that the hydrophilic bilayer modification significantly enhanced the dispersion stability of Gd_2_O_3_ nanoparticles in aqueous solutions. The remarkable improvement in dispersibility is primarily attributed to the presence of hydrophilic polar groups—namely, sulfonic acid groups in SIT and amine groups in M2070—on the nanoparticle surfaces, which effectively promote interactions with water molecules. Moreover, the introduction of these bilayers substantially reduced the water contact angle and lowered the zeta potential, further indicating enhanced hydrophilicity and colloidal stability. Thermogravimetric analysis (TGA) verified the presence and thermal stability of the dual organic coating, highlighting its robustness at moderate temperatures.

Consequently, the modified Gd**_2_**O**_3_**@SIT-M nanoparticles exhibit excellent aqueous dispersion stability, remaining uniformly dispersed without aggregation or sedimentation even after extended periods of storage. Such outstanding dispersibility in aqueous and polymer matrices positions these materials as promising candidates for incorporation into functional composites, especially in neutron shielding applications. This novel surface modification strategy not only ensures improved material compatibility but also provides a solid foundation for developing advanced neutron-shielding materials with enhanced processability and performance.

## Data Availability

Dataset available from the corresponding author upon request.

## Supplementary Materials

The following supporting information can be downloaded at: https://www.mdpi.com/article/10.3390/nano15181421/s1, Figure S1: The EDS mapping of Gd_2_O_3_@SIT containing the elements of C, O, Si, and Gd; Figure S2: XRD patterns of Gd_2_O_3_, Gd_2_O_3_@SIT and Gd_2_O_3_@SIT-no Cation; Figure S3: XRD patterns of Gd_2_O_3_, Gd_2_O_3_@KH550 and Gd2O3@KH550-no Cation; Figure S4: XRD patterns of Gd_2_O_3_, Gd_2_O_3_@KH560 and Gd_2_O_3_@KH560-no Cation; Figure S5: Rietveld refinement of the XRD patterns for the synthesized samples; Figure S6: Photographs of aqueous stabilities of 10.0 mg/mL of long-standing Gd_2_O_3_@SIT-M. Table S1: The lattice parameters of the Sample 1 (Gd_2_O_3_) and Sample 2 (Gd(OH)_3_ and Gd_2_O_3_) according to the XRD refinement analysis.
