# Supplementary material for: Hydrophilic Modification of Gadolinium Oxide by Building Double Molecular Structures
Source: Nanomaterials (Basel). 2025 Sep 16;15(18):1421. doi: 10.3390/nano15181421 (PMC12472387; doi:10.3390/nano15181421)
Supplement: Supplementary file 1 [file nanomaterials-15-01421-s001.zip › nanomaterials-3825811-supplementary.pdf]

Supporting Information for

Hydrophilic modification of gadolinium oxide by building double molecular structures

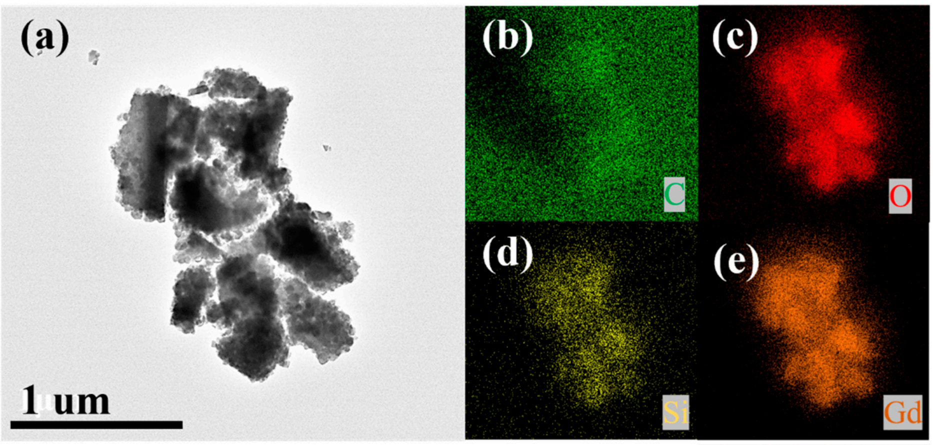

Figure S1. The EDS mapping of Gd_2O_3@SIT containing the elements of C, O, Si, and Gd.

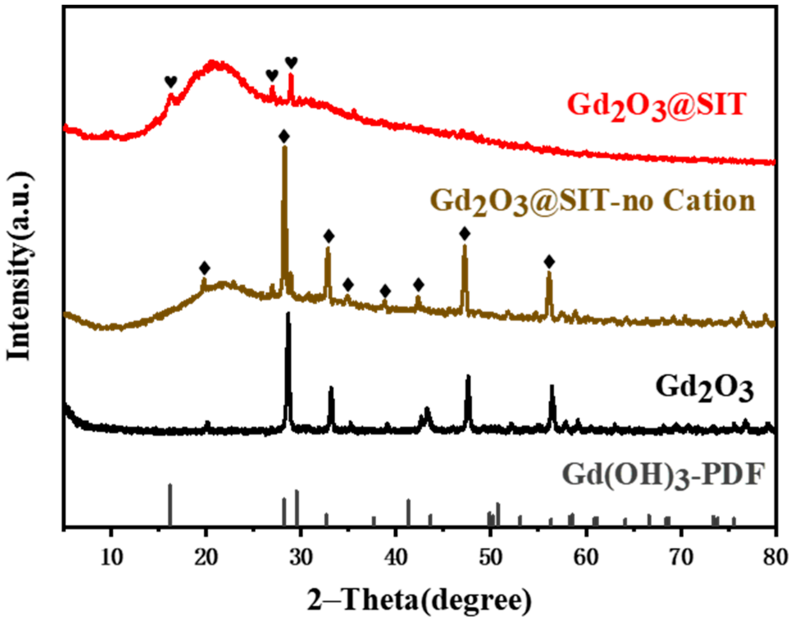

Figure S2. XRD patterns of Gd_2O_3, Gd_2O_3@SIT and Gd_2O_3@SIT-no Cation.

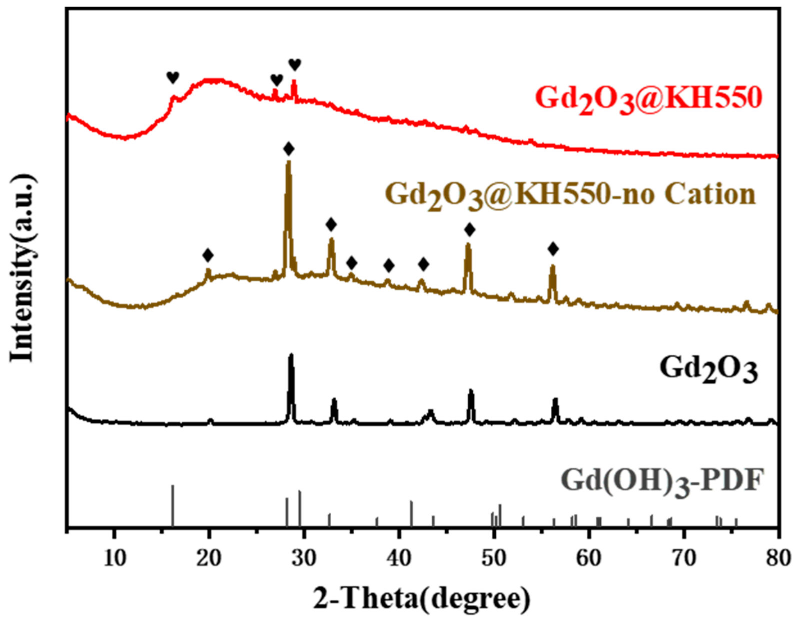

Figure S3. XRD patterns of Gd_2O_3, Gd_2O_3@KH550 and Gd_2O_3@KH550-no Cation.

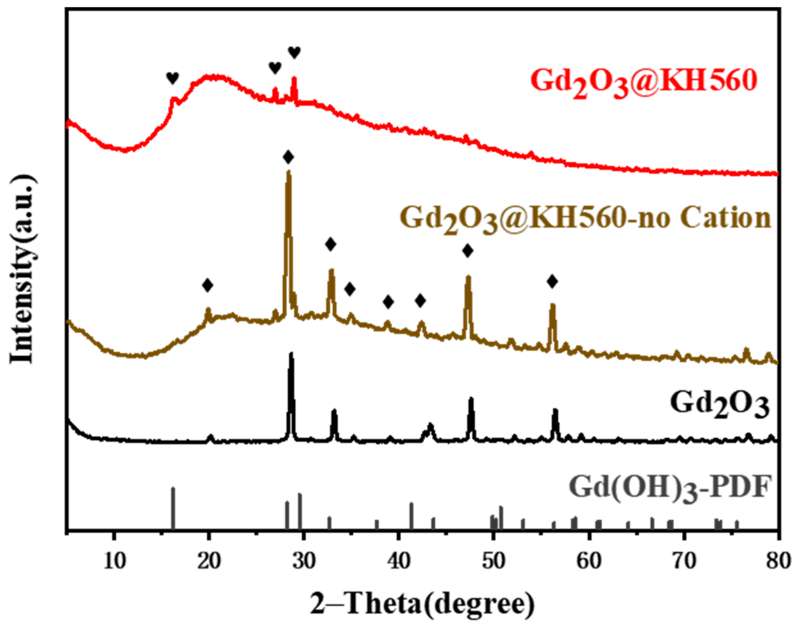

Figure S4. XRD patterns of Gd_2O_3, Gd_2O_3@KH560 and Gd_2O_3@KH560-no Cation.

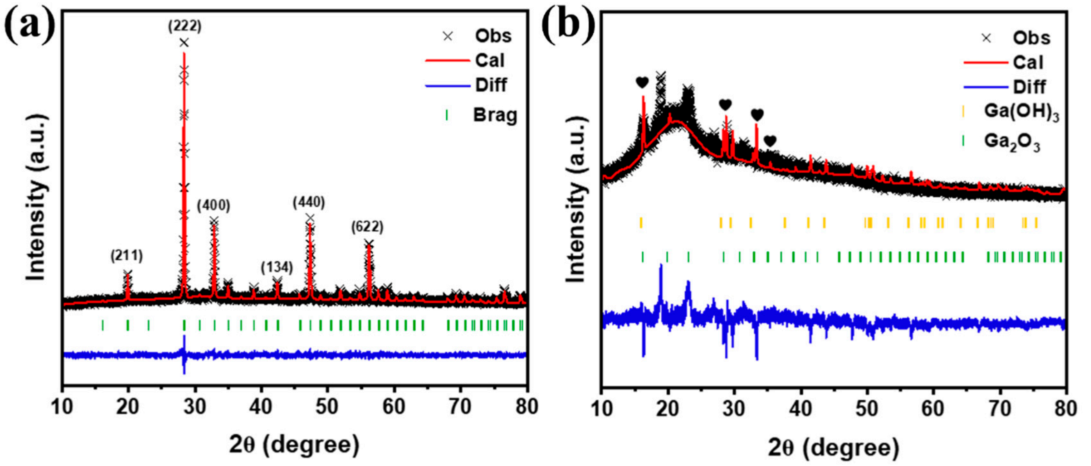

Figure S5. Rietveld refinement of the XRD patterns for the synthesized samples. (a) Gd_2O_3 and (b) $Gd(OH)_3$ and Gd_2O_3. The experimental data are shown as black circles, the calculated profiles are represented by the red solid lines, and the difference curves are displayed in blue at the bottom. The vertical tick marks indicate the Bragg reflection positions for the corresponding crystalline phases.

Table S1. The lattice parameters of the Sample 1 (Gd_2O_3) and Sample 2 ($Gd(OH)_3$ and Gd_2O_3) according to the XRD refinement analysis.

Sample Name		Space group	Crystal System	Lattice parameters						Agreement Factors		
				a [Å]	b [Å]	c [Å]	A [°]	B [°]	Γ [°]	R_p [%]	R_{wp} [%]	χ^2
1	Gd_2O_3	Ia-3	Cubic	10.8099	10.8099	10.8099	90	90	90	3.72	4.66	1.13
2	Gd_2O_3	Ia-3	Cubic	10.8008	10.8008	10.8008	90	90	90	24.02	25.94	13.50
	$Gd(OH)_3$	P63/m	Hexagonal	6.3291	6.3291	3.6126	90	90	120			

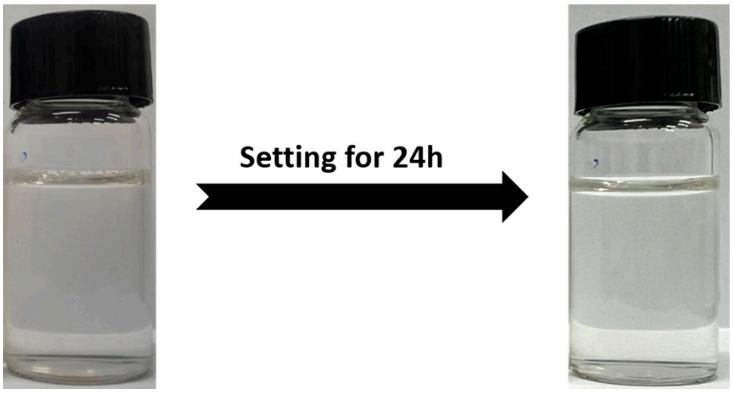

Figure S6. Photographs of aqueous stabilities of 10.0 mg/mL of long-standing Gd_2O_3@SIT-M.
